# Comparison of clinical effectiveness and subsequent fertility between hysteroscopic resection and vaginal repair in patients with cesarean scar defect: a prospective observational study

**DOI:** 10.1186/s12958-023-01169-4

**Published:** 2023-12-11

**Authors:** Guoxia Yang, Jiamin Wang, Yajie Chang, Yuqing Chen

**Affiliations:** 1 Reproductive Medical Center, The First Affiliated Hospital of Sun Yat-Sen University, Guangzhou, China; 2 Department of Obstetrics and Gynecology, The First Affiliated Hospital of Sun Yat-Sen University, Zhongshan 2 Road, Guangzhou, China; 3 Guangdong Provincial Clinical Research Center for Obstetrical and Gynecological Diseases, Guangzhou, China; 4 Reproductive Medical Center, The Six Affiliated Hospital of Sun Yat-Sen University, Guangzhou, China

**Keywords:** Cesarean scar defect, Hysteroscopy, Vaginal, Subsequent fertility

## Abstract

**Objective:**

To evaluate the clinical effectiveness and pregnancy rate after hysteroscopic resection (HR) and/or vaginal repair (VR) in patients with cesarean scar defect (CSD).

**Methods:**

This prospective observational study enrolled 191 patients who received CSD surgery in the First affiliated hospital of Sun Yat-sen University between September 2019 to February 2022 (96 in HR and 95 in VR, respectively). Patient follow-up were performed three months after surgery in both groups by transvaginal ultrasound to confirm the presence of fluid in the niche, along with the resolution of prolonged menses at the same time. The primary outcome was the clinical effectiveness between HR and VR, identified by the resolution of prolonged menses.

**Results:**

The rates of niche-fluid disappearance (70.1% *vs* 60.2%, *P* = 0.176) and prolonged menses resolution (74.8% *vs* 80.0%, *P* = 0.341) were comparable for HR and VR. A subgroup analysis for niche size revealed that HR provides patients with small niche a more favorable rate of menstrual resolution compared to VR (size of niche ≤ 15 mm^2^, ^a^OR = 3.423, 95% confidence interval [CI] 1.073–10.918), but patients with large niche experience a lower rate of resolution compared to VR (size of niche > 25 mm^2^, ^a^OR = 0.286, 95% CI 0.087- 0.938). During follow-up, 41 patients who wanted to conceive became pregnant. Kaplan–Meier estimates of the cumulative probability of pregnancy at 12 months and 24 months were 47.1% (95% CI: 34.5%, 58.8%) and 63.8% (95% CI: 52.5%, 72.9%), respectively. The median pregnancy time was 22 months (95% CI: 14.2, 29.8) after VR and 12 months (95% CI: 8.3, 15.7, Gehan-Breslow-Wilcoxon *P* = 0.021) after HR. Among patients with subsequent infertility, 31.6% achieved pregnancy by unassisted mode and 29.8% by IVF/ICSI. Moreover, among patients with previously failed IVF/ICSI treatment, 60% (12/20) obtained pregnancy, including 71.4% (10/14) after HR and 33.3% (2/6) after VR.

**Conclusions:**

Hysteroscopic resection is as effective as vaginal repair at relieving symptoms of CSD-associated prolonged menses. Hysteroscopic resection is the modality of choice with an improvement in prolonged menses for small niche, while vaginal might be considered for a large niche. Furthermore, surgical intervention could improve the clinical pregnancy rate of CSD patients. All of these provide evidence for the individualized management of CSD.

**Supplementary Information:**

The online version contains supplementary material available at 10.1186/s12958-023-01169-4.

## Introduction

There has been a marked increase in cesarean section (CS) rate globally in the past few years [1], with rates as high as 44.5% in 2020 in China [2]. Recently, the long-term complication of CS, cesarean scar defect (CSD), has become an undesired consequence, owing to the fact that 76.4% of CSD patients suffer from prolonged menses [3]. Moreover, subsequent fertility may be impaired, with an estimated risk of infertility ranging from 4% to 19% [4]. In addition, patients with CSD may be associated with adverse IVF/ICSI outcomes [5].

Currently, the literature clearly shows that surgical repair of CSD can improve the symptoms of prolonged menses. Several systematic reviews showed that the success ranges for treatment of associated prolonged menses vary greatly, with hysteroscopic resection showing an improvement of 59%–100% [6], and vaginal repair showing 89%–93.5% recovery [7]. Therefore, it is controversial regarding the suitable surgical treatment of CSD. However, most studies settling this debate are limited by case series [8], without control group [9], retrospective design [10], and inconsistency of results across studies [11]. There is clear need for more and better researches that help us justify the implementation of these surgical treatments for the prolonged menses. Furthermore, it is suggested that these interventions are helpful to improve secondary infertility after a CS and could be supplementary to or substitute assisted reproductive technology (ART) [12]. However, the effect of these interventions on fertility outcomes remains unclear [13]. A review published in 2023 provided an overview of available literature on reproductive outcomes after surgical treatment, the investigators concluded that there was not enough high-quality evidence of the benefits on fertility outcomes after surgical correction of CSD [11].

Thus, we conducted this prospective study to assess the clinical effectiveness of hysteroscopic resection (HR) and vaginal repair (VR) for patients with CSD in a large series (*n* = 191). Furthermore, we aimed to provide evidence on the effectiveness and safety of surgical interventions for CSD in relation to patients diagnosed with or without infertility and their effect on obstetric outcomes.

## Materials and methods

### Patients

This prospective observational study enrolled patients with CSD who were admitted to the Department of Obstetrics and Gynecology of the First Affiliated Hospital of Sun Yat-sen University in China between September 2019 to February 2022 (NCT04096677, www.clinicaltrials.gov).

The key inclusion criteria were as follows: diagnosed as CSD by ultrasound; presented 3 days longer menses than baseline, no previous treatment for CSD, age < 40y at the time of enrollment. The diagnosis of CSD was confirmed by transvaginal ultrasound (TVU) according to the European Niche Taskforce [14]. The size of niche was calculated using the formula: base times height divided by two according to the previous study (Supplementary Fig. 1). Thus, the niche was divided into three grades as follows: Grade 1 (less than 15 mm^2^, small niche), Grade 2 (16 to 25 mm^2^, medium niche), and Grade 3 (greater than 25 mm^2^, large niche) [15].

The key exclusion criteria were as follows: intrauterine adhesions, endocrine disorder, abnormal uterine bleeding explained by other diseases, such as endometrial hyperplasia, submucosal fibroids, endometrial cancer and so on.

**Fig. 1:**
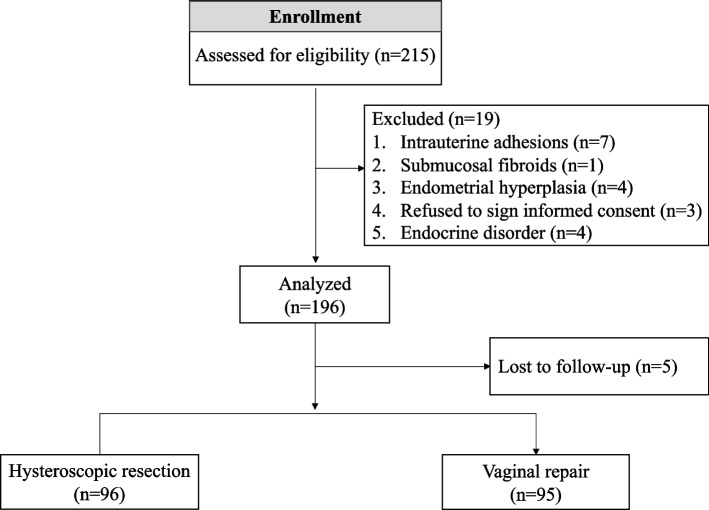
Flow chart of study

All patients consented to participate after receiving clear information on the advantages and disadvantages of both HR and VR. Patients were assigned to either HR or VR according to their preference. Finally, a total of 191 patients were analyzed prospectively. The study flowchart was shown in Fig. 1.

The study was approved by the Institutional Review Board of the First Affiliated Hospital of Sun Yat-Sen University (NO.2020–242).

### Procedure

CSD repair was performed during proliferative phase (day 9–11 of the menstrual cycle). HR and VR was performed by the same senior surgeons respectively. In addition, the patients attempting pregnancy received endometrium biopsy for Syndecan-1 (CD138). Patients with CD138^+^/HPF ≥ 5 were diagnosed as chronic endometritis (CE), and they were treated with oral antibiotics after the CSD repair in accordance with prior research [16].

### Hysteroscopic resection

A hysteroscopic instrument (STORZ, Germany) was used during the operation, and physiological saline was employed as the distention medium. All patients underwent diagnostic hysteroscopy to confirm cervical canal, niche, uterine cavity, and fallopian tube opening (Supplementary Fig. 2A). The cervix was dilated to 10 mm using a Hegar dilator. Afterward, the inferior edges of the CSD were resected using a cutting loop to enable visualization of the niche, creating a slope between the cervix and the niche's bottom (Supplementary Fig. 2B). Next, in order to avoid thermal damage to the surrounding tissues, the endometrium and surficial vessels on the thinnest part of the niche were coagulated using a ball electrode at low power, quickly rolling or spot electrocoagulation, and not rolling back and forth (Supplementary Fig. 2C-D). All operations were carried out under an inflation pressure of 100–120 mmHg and a flow rate of 100–150 mL/min.

### Vaginal repair

After conventional bladder catheterization, a vaginal retractor was inserted to visualize the cervix uteri, the anterior lip of the cervix held with grasping forceps, and Adrenaline (1:2000) was injected into the vesico-cervical junction to create separation via hydraulic pressure. A transverse incision on the anterior vaginal wall was used to enter vesico-cervical space. The bladder was manually pushed upward to the vesico-peritoneal reflection, and the abdominal cavity was entered to expose the site of the previous uterine incision. Unipolar scissors were used to trim the scar and its surrounding tissue. 1–0 absorbable suture was used to repair the cutting edge of the uterus, and 2–0 absorbable suture was used to reapproximated the muscular layer around the incision. An additional layer of continuous or interrupted sutures was added to strengthen the area, then a Hegar dilator was inserted through the cervix into the scar to confirm that the defect had been repaired. After confirming no active bleeding, the bladder peritoneum and vaginal vault were reapproximated using 2–0 absorbable suture.

### Outcomes

The primary outcome was the clinical effectiveness between HR and VR, identified by the resolution of prolonged menses. Patient follow-up were performed personally three months after surgery in both groups by TVU to confirm the presence of fluid in the niche, along with the resolution of prolonged menses at the same time. The impact of the surgery on the symptom of prolonged menses were assessed by a score system as follows: (I) 2 points: complete resolution of prolonged menses. (II) 1 point: shortened reduction of more than 50% in prolonged menses. (III) 0 point: no obvious change of menstruation postoperatively. 2 and 1 point were recorded as improvement, and 0 point was regarded as fail. Patients were advised to conceive one month after HR, but they were recommended to wait at least six months after VR. The subsequent pregnancy outcomes were contacted by telephone interview thereafter, including the method of conception, time to pregnancy, and prenatal complications (placenta previa, placental increta, placenta adhesion, postpartum hemorrhage, and uterine rupture).

### Sample size

According to the clinical effectiveness of HR and VR published in the literature, we estimated that the clinical effectiveness of HR and VR is about 75.0% and 90.0%, respectively. Thus, a minimal of 107 patients per group were recruited, considering a two-sided alpha of 0.05, power of 80% and missed follow-up rate of 10%.

### Statistical analysis

The data were analyzed using SPSS version 22.0 (SPSS Inc, Chicago, IL, USA), with a significance level of *P* < 0.05 for all tests. Values were expressed as mean ± standard deviation or median for continuous variables and as proportion (%) for categorical variables. Single-variable analysis was performed by t-tests, and the Chi-Squared test was performed for categorical variables. An exploratory stratified analysis was performed, and the risk factors were adjusted by logistic regression model. Cumulative probabilities of pregnancy over time were estimated with the Kaplan–Meier method to account for varying follow-up times. The Gehan-Breslow-Wilcoxon test was used to test whether study outcomes were significantly different between the two groups.

## Results

### General characteristics of the study population

Of the 215 patients who underwent surgery for CSD, a total of 191 patients were included in the analysis. The baseline characteristics of the two groups are shown in Table 1. There was no significant difference between the two groups in terms of age, BMI, number of previous cesarean sections, area of niche, and uterine position.

**Table 1:** Baseline characteristics of patients included in this study

Characteristic	Hysteroscopic resection (*n* = 96)		Vaginal repair (*n* = 95)	*P* value
Age (y)	34.1 ± 4.613		33.2 ± 4.125	.174
BMI (kg/m^2^)	20.6 (19.1–23.0)		20.7 (19.2–22.1)	.896
Gravidity	1 (1–2)		2 (1–2)	.354
Parity	1 (1–2)		2 (1–2)	.122
Abortion	1 (0–2)		0 (0–1)	.322
**Number of previous CS (%)**				.068
1	50 (52.1)		37 (38.9)	
≥ 2	46 (47.9)		58 (61.1)	
**Symptoms before treatment (%)**				
Prolonged menses	96(100)		95(100)	-
Infertility	42(37.5)		28(26.3)	.093
Niche-fluid by Ultrasound (%)	87(90.6)		83(87.4)	.472
**Area of niche (mm**^**2**^**) (%)**				.211
Grade 1	36 (37.5)		26 (27.7)	.211
Grade 2	17 (17.7)		25 (26.6)	
Grade 3	43 (44.8)		43 (45.7)	
**Uterine position (%)**				.565
Anteflexion	44 (45.8)		47 (50)	
Retroflexion	52 (54.2)		47 (50)	
**CD138 + /HPF ≥ 5 (%)**	25/76(32.9)		15/57 (26.3)	.413
**Surgical indicators**				
Hospital stays (d)	2 (1–2)		3 (3–5)	< .001
Operative time (min)	20 (15–30)		45 (30–65)	< .001
Bleeding volume (mL)	5 (5–10)		30 (15–50)	< .001
Fever (> 37.3 ℃)	0		23 (24.2)	< .001
Perforation	0		0	
**Postoperative menstruation (%)**				.341
2 points	49 (48.6)		33 (38.1)	
1 point	22 (26.2)		42 (41.9)	
0 point	23 (25.2)		19 (20.0)	
**Disappearance of niche-fluid (%)**	61 (70.1)		50 (60.2)	.176

Data are reported as mean ± standard deviation, as n (percentage), or as median (interquartile range, IQR).CS = cesarean section. In 21 patients, information on the niche fluid after surgery was missed

### Clinical effectiveness of HR and VR for CSD

The median operative time, length of hospital stays, and bleeding volume were significantly shorter in the group of HRs than VRs (all *P* < 0.001, Table 1). No evident complications including bladder trauma occurred in both groups. There was no significant difference in the resolution of prolonged menses between the two groups (74.8% vs. 80%, *P* > 0.05, Table 1). Additionally, the niche-fluid disappeared in 61 (70.1%) patients after HR and 50 (60.2%) patients after VR, and no significant differences were observed between the two groups (*P* > 0.05).

Subgroup analyses regarding the effects of niche size on the association between surgical approach and resolution of prolonged menses are shown in Table 2. For patients with small niche, HR provide a more favorable outcome in menstruation resolution (OR = 3.429, 95% confidence interval [CI] 1.106–10.631, *P* = 0.029), despite the adjustment for age, uterine position and number of CS (^a^OR = 3.423, 95% CI 1.073–10.918, *P* = 0.038). In contrast, HR was associated with a lower rate of resolution for prolonged menses in patients with large niche compared with VR (OR = 0.294, 95% CI 0.094–0.904, *P* = 0.029), and adjusted OR was 0.286 (95% CI 0.08–0.938, *P* = 0.039). No significant difference was observed for medium niche (^a^OR = 0.840, 95% CI 0.138–5.121,* P* = 0.85).

**Table 2:** Stratification analysis regarding the effects of niche’s size on the association between surgical approach and resolution of prolonged menses

	Factor	OR	P	^a^ OR	**P**
**Grade 1**					
	**Surgery approach**				
	HR	3.429 (1.106–10.631)	0.029	3.423(1.073–10.918)	.038
	VR	Reference			
**Grade 2**					
	**Surgery approach**				
	HR	0.848 (0.165–4.374)	0.844	0.840(0.138–5.121)	.850
	VR	Reference			
**Grade 3**					
	**Surgery approach**				
	HR	0.294 (0.094–0.904)	0.029	0.286 (0.087–0.938)	.039
	VR	Reference			

^*a*^*OR* were calculated by logistic regression analysis with adjustments of age, uterine position and number of CS. OR = odds ratio, ^a^OR = adjusted odds ratio, 95% CI = 95% confidence interval

*HR* hysteroscopic resection, *VR* vaginal repair

### Subsequent clinical pregnancy of patients with CSD after surgical treatment

**Fig. 2:**
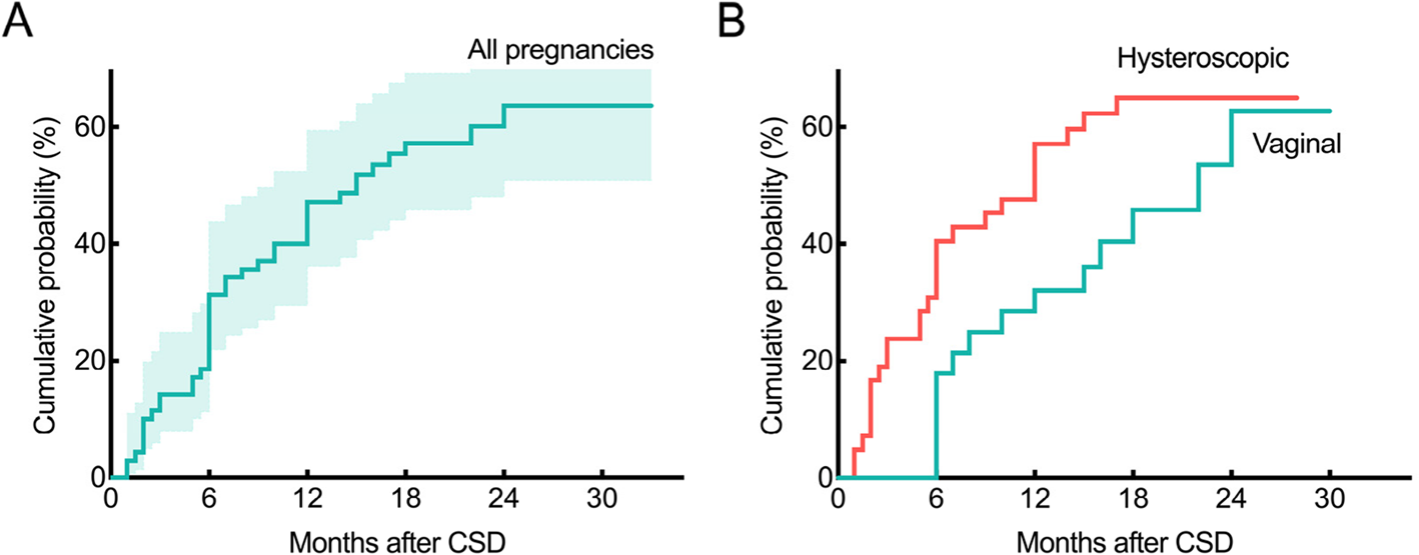
**A** Overall cumulative probabilities of pregnancy after CSD in patients who wish to conceive. **B** Cumulative probability of pregnancy was compared between VR (green line) and HR (red line) in patients who wish to conceive. Analyses were performed with Kaplan–Meier estimates, and Gehan-Breslow-Wilcoxon tests

The study population included 70 patients who wanted to conceive, and seven patients were lost to follow-up (10.0%). The median follow-up time was 22 months (range 3–33). Kaplan–Meier estimates of the cumulative probability of pregnancy at 12 months and 24 months were 47.1% (95% CI: 34.5%, 58.8%) and 63.8% (95% CI: 52.5%, 72.9%, Fig. 2A), respectively. The estimates of the cumulative probability of pregnancy at 12 months were 32.1% (95% CI: 8.7%, 51.0%) after VR and 57.1% (95% CI: 42.9%, 68.9%, Fig. 2B) after HR. The median pregnancy time was 22 months (95% CI: 14.2, 29.8) after VR and 12 months (95% CI: 8.3, 15.7, Gehan-Breslow-Wilcoxon *P* = 0.021) after HR.

All these pregnancies resulted in 33 live births, including 24 after HR and 9 after VR. Almost all births are by planned CS, with the exception of one vaginal birth after VR. No uterine rupture was observed in all pregnancies. In addition, one patient in each group developed an ectopic pregnancy, without cesarean scar pregnancy. Five patients had perinatal complications including placenta previa, placenta accreta and placental increta after surgical treatment, 4 after HR and 1 after VR (Table 3).

**Table 3:** Pregnancy outcomes of patients with fertility desire

	**Hysteroscopic resection (*n* = 39)**	**Vaginal repair (*n* = 24)**	***P* value**
Clinical Pregnancy (%)	27 (69.2)	14 (58.3)	.341
Live birth (%)	24 (61.5)	9 (37.5)	.064
Missed abortion (%)	2 (7.4)	4 (28.6)	.157
Ectopic pregnancy (%)	1 (3.7)	1 (7.1)	.728
Time Interval (months)	6.6 ± 4.8	11.2 ± 6.8	.016
**Delivery mode (%)**			.333
CS	24 (100)	13 (92.9)	
Vaginal	0	1 (7.1)	
**Pregnancy mode (%)**			.113
Unassisted	18 (46.2)	16 (66.7)	
IVF/ICSI	21 (53.8)	8 (33.3)	
**Preterm birth (%)**	2 (8.3)	1 (11.1)	.808
**Pregnancy complications (%)**			.842
Placenta previa	1 (2.6)	0 (0.0)	
Placenta accreta	2 (5.1)	1 (4.2)	
Placenta increta	1 (2.6)	0 (0.0)	

*CS* cesarean section, 7 patients missed the value of pregnancy outcome

**Fig. 3:**
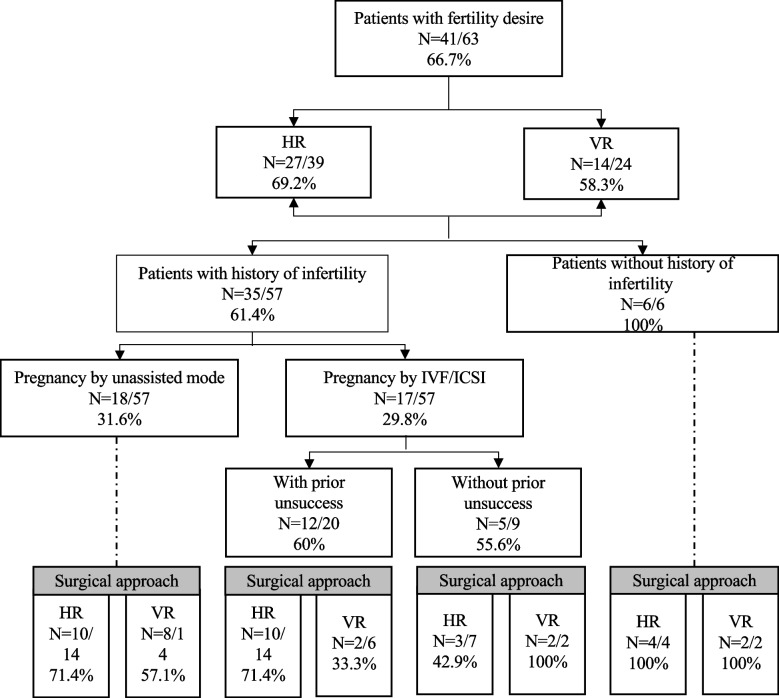
Flow diagram of patients with fertility desire after surgery

Among 63 patients attempting pregnancy, 57 patients had been trying to conceive for > 1 year by the time that they were referred to our department, and 31.6% of them conceived with unassisted mode, and 29.8% conceived after IVF/ICSI treatment. What’s more, among patients with previously failed ART before the surgical intervention, 60% conceived after surgery, and the patients were more likely to achieve pregnancy after HR, although not statistically significant (71.4% vs 33.3%, *P* = 0.111, Fig. 3).

## Discussion

This was a prospective study comparing clinical effectiveness of HR and VR for patients with CSD in a large series. Our findings showed that HR was effective at improving symptoms of prolonged menses and eliminating the CSD-associated niche-fluid, similar to VR. Furthermore, we tentatively put forward that size of niche was associated with choosing different management of CSD. More importantly, surgical management could significantly improve the subsequent fertility of CSD patients with or without infertility. These findings may be useful for patient counseling and medical decision-making.

CSD has severely affected the quality of life of patients [17] and increased the risk of adverse outcomes in patients with re-pregnancy. However, there is a lack of high-quality evidence sustaining the best surgical approach and criteria, as well as the potential benefits of surgical repair on fertility, which were limited by the lack of uniformity in CSD diagnosis, inconsistency in indication for surgery [18], missing features of the niche [19], and small case number [20]. In this prospective study, all patients presented prolonged menses before surgery, and we found that HR induced comparable effectiveness to VR, including relieving symptoms of prolonged menses and removing the fluid in the niche [21], which was in agreement with a recent meta-analysis [21]. In addition, we offered some evidence for the safety of HR. In contrast, VR may be more likely to result in a postoperative infection because of the relatively difficult suture, the large wound, and other factors. Hence, HR may be a safer and less invasive option in patients with CSD.

In the stratified analysis, we found that patients with large niche had poorer menstruation improvement after HR, compared with VR. This may be due to the possibility that a large niche could lead to poorer contractility owing to the myometrial defect, convinced by the fact that increasing symptoms were associated with large defects [3]. In such cases, VR is able to restore the thickness of the anterior uterine wall [22]. Deng et al. observed the mean residual myometrial thickness (RMT) increased from 2.25 mm to 5.30 mm in a series of 183 patients after surgery [18], proving adequate reinforcement of the myometrium. In contrast, patients with small niche had significant improvement following HR. It might be associated with enough residual muscle in a small niche, and only by cutting the lower edge can facilitate fluid drainage during HR. In contrast, VR will disrupt the normal healing process, and the defect may develop again after surgical repair [23]. Our findings regarding the selection of a surgical approach depending on the size of niche provide individualized management of CSD. ﻿ However, the focus on improved symptoms following VR in patients with large niche should be balanced more clearly against increased operative risk. More evidence is needed to further justify choosing a surgical approach based on the size of niche.

Many studies have established that CSD may exert a detrimental effect on fertility [5, 24]. A study published recently showed that patients with niche had reduced implantation rate, clinically pregnancy rate and live birth rate in IVF/ICSI treatment [5]. It was proposed that the persistence of blood or mucus in the defect worsen the environment for embryo implantation [4]. And in the current study, we found that pregnancy is more likely to occur in patients without niche-fluid after surgery (82.9% vs. 54.5%, *P* < 0.05, Supplemental Table 1). Additionally, we discovered that 30% of patients with CSD had CE diagnoses (26.3% in VR and 32.9% in HR, Table 1), which was greater than the 15.7% in patients without intrauterine disorders reported by Kuroda et al. [25]. Wei et al. also suggested that CSD may increase the risk of CE [26], which may affect the endometrium receptivity [27].

Regarding the patients with secondary infertility, we found that the pregnancy rate after CSD repair was improved in both groups. Although our findings were in accordance with those of previous studies [13, 28], the strengths of our study should be pointed out. Firstly, there was no information on the mode of pregnancy in most studies. We expected to evaluate the impact of surgery in the whole population, not restricted to patients with IVF/ICSI [29]. Secondly, none of the selected studies reported the subsequent pregnancy outcome after surgery in patients who had previously failed ART. In our study, 60% (12/20) patients with prior unsuccess achieved pregnancy after surgery (10 after HR and 2 after VR), although not statistically significantly, which may be associated with the small sample size. Additionally, the median pregnancy time was relatively shorter in HR as expected, considering that patients were advised not to pregnancy within 6 months after VR in the current study. This would be a reason why there was a delayed time to pregnancy (also shown in Fig. 2) in VR as compared to HR. Previous studies pointed out that scar healing achieves a relatively stable state 6 months postoperatively after CS [30]. Therefore, HR may be beneficial for patients who intend to conceive as soon as possible after surgery, such as those with advanced age or poor ovarian reserve, especially with the opening of China’s three-child policy. Our study demonstrated the additional value of niche resection for fertility therapy, especially for HR.

In response to the consensus, HR is not recommended when the RMT is < 3 mm [31]. In our study, RMT of the niche was not measured. We think that the RMT measurement may be inaccurate, considering the residual myometrium may be compressed by the pouch. Furthermore, variations in technique might impact the rationale for choosing HR for RMT < 3 mm. For example, the surgical technique to manage CSD described by Italian team consists of excising one or both the cephalad and caudal portions using a threshold of greater than 4 mm, in order to restore continuity between the uterine cavity and the internal orifice of the cervix [32, 33]. In our study, only the inferior edge of the CSD was resected to enable visualization of the niche, and for the niche bottom, the thinnest part, only the endometrium and surficial vessels are coagulated using a ball electrode. In accordance with Zeller et al., HR appears to be an effective strategy for managing CSD with RMT < 3 mm [9]. Moreover, Tsuji et al. showed an increase in RMT from 2.1 mm to 4.2 mm [34, 35], while Tanimura et al. discovered that RMT remained unchanged after HR in a small series [36].

However, it should be mentioned that 4 patients had perinatal complications including placenta previa, placenta accreta and placental increta after HR. In fact, the patient with placenta previa had a history of the condition in a previous pregnancy, according to historical records. The other patient who encountered placenta increta during pregnancy had a history of two previous CSs and three abortions for early embryonic arrest. Therefore, the safety of HR for future pregnancy needs to be interpreted cautiously due to the small sample size.

A particular strength of our study was the prospective evaluation of a larger series, and the same senior specialist performed both HR and VR separately, controlling for any bias imposed by the surgeon. However, there were several limitations in our study. Firstly, the number of participants was relatively small; therefore, validation in a larger population is required. The second is related to the bias of no randomized control. In the future, more randomized controlled trials are needed to confirm our results. Thirdly, our study considered only niche’s depth and width, ignoring other parameters for clinical effectiveness assessment, such as RMT, the distance between the niche and the ectocervix, and so on. In addition, it is meaningful to follow up on the long-term clinical outcomes and reproductive prognosis of patients with CSD after surgical management.

## Conclusion

In conclusion, hysteroscopic resection is as effective as vaginal repair at relieving symptoms of CSD-associated prolonged menses. Hysteroscopic resection is the modality of choice with an improvement in prolonged menses for small niche, while vaginal might be considered for a large niche. Furthermore, surgical intervention could improve the clinical pregnancy rate of CSD patients. All of these provide evidence for the individualized management of CSD.

### Supplementary Information

**Additional file 1:** **Supplementary Figure 1.** Schematic presentation of ultrasound measurement of the niche. a: the base directed to the posterior wall of the cervical canal b: the apex pointing to the anterior wall of the niche. **Supplementary Figure 2.** Hysteroscopic resection. A: Hysteroscopic view of a defect at the anterior uterine wall. B: Resection of the lower rim using a resectoscope. C: Coagulation of the niche’s surface. D: Hysteroscopic view after resection. **Supplemental Table 1.** Clinical characteristics of patients attempting pregnancy.

## Data Availability

The original data presented in the study are included in the article/supplementary material. Further inquiries can be directed to the corresponding author.

## Abbreviations

CSD: Cesarean scar defect
VR: Vaginal repair
HR: Hysteroscopic resection
CS: Cesarean section
ART: Assisted reproductive technology
TVU: Transvaginal ultrasound
CD138: Syndecan-1
CE: Chronic endometritis
RMT: Residual myometrial thickness
