# Supplementary material for: Comparison of clinical effectiveness and subsequent fertility between hysteroscopic resection and vaginal repair in patients with cesarean scar defect: a prospective observational study
Source: Reprod Biol Endocrinol. 2023 Dec 11;21:119. doi: 10.1186/s12958-023-01169-4 (PMC10712091; doi:10.1186/s12958-023-01169-4)
Supplement: Supplementary file 1 — Additional file 1: Supplementary Figure 1. Schematic presentation of ultrasound measurement of the niche. a: the base directed to the posterior wall of the cervical canal b: the apex pointing to the anterior wall of the niche. Supplementary Figure 2. Hysteroscopic resection. A: Hysteroscopic view of a defect at the anterior uterine wall. B: Resection of the lower rim using a resectoscope. C: Coagulation of the niche’s surface. D: Hysteroscopic view after resection. Supplemental Table 1. Clinical characteristics of patients attempting pregnancy. [file 12958_2023_1169_MOESM1_ESM.docx]

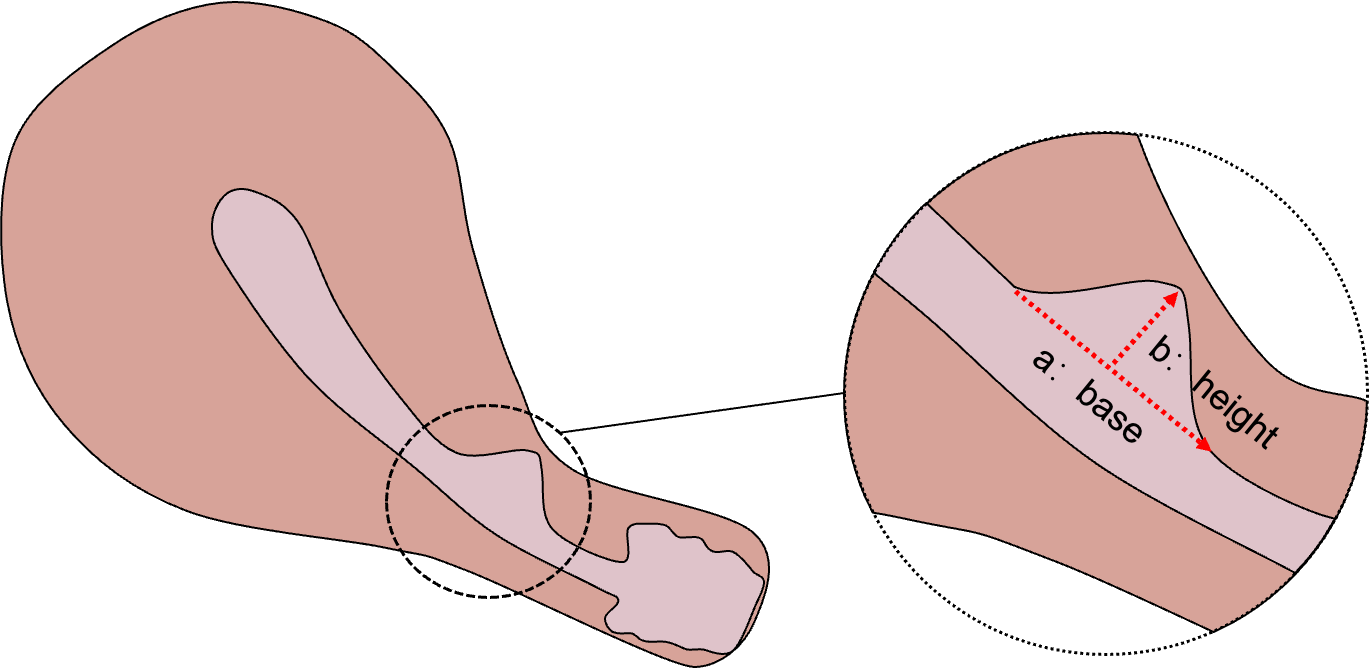


**Supplementary Figure 1** Schematic presentation of ultrasound measurement of the niche. a: the base directed to the posterior wall of the cervical canal b: the apex pointing to the anterior wall of the niche

**Supplementary Figure 2** Hysteroscopic resection. **A**: Hysteroscopic view of a defect at the anterior uterine wall. **B**: Resection of the lower rim using a resectoscope. **C**: Coagulation of the niche’s surface. **D**: Hysteroscopic view after resection

| **Supplemental Table 1** Clinical characteristics of patients attempting pregnancy. | | | |
| --- | --- | --- | --- |
| **Parameters** | **Pregnancy(n=41)** | **Non-pregnancy(n=22)** | **P value** |
| **Age (y)** |  |  | .001 |
| < 35 | 32 (78.0) | 8 (36.3) |  |
| ≥ 35 | 9 (22.0) | 14 (63.6) |  |
| **Number of previous CS (%)** | |  | .517 |
| 1 | 36 (87.8) | 18 (81.8) |  |
| ≥ 2 | 5 (12.2) | 4 (18.2) |  |
| **Area of niche (%)** |  |  | .018 |
| Grade I | 14 (34.1) | 1 (4.5) |  |
| Grade II | 9 (22.0) | 10 (45.5) |  |
| Grade III | 18 (43.9) | 11 (50.6) |  |
| **Postoperative menstruation (%)** |  |  | .226 |
| Improvement | 38 (92.7) | 18 (81.8) |  |
| Fail | 3 (7.3) | 4 (18.2) |  |
| **Type of conception (%)** |  |  | .321 |
| Unassisted | 24 (58.5) | 10 (45.5) |  |
| ART | 17 (41.5) | 12 (54.5) |  |
| **Surgery approach (%)** |  |  | .378 |
| HR | 27 (65.9) | 12 (54.5) |  |
| VR | 14 (34.1) | 10 (45.5) |  |
| **Niche-fluid postoperatively (%)** |  |  | .016 |
| Absence | 34 (82.9) | 12 (54.5) |  |
| Persistent | 7 (17.1) | 10 (45.5) |  |
| *Notes:* CS = cesarean section, ART = Assisted Reproductive Technology, | | | |
